# Supplementary material for: The impact of different standing positions on gluteus medius activation and lumbar lordosis in LBP-developers during prolonged standing
Source: PLoS One. 2025 Feb 11;20(2):e0317291. doi: 10.1371/journal.pone.0317291 (PMC11813126; doi:10.1371/journal.pone.0317291)
Supplement: S1 Table — (DOCX) [file pone.0317291.s001.docx]

| \| **Examiner score** \| \| --- \| | \| **Cues for examiner** \| \| --- \| |
| --- | --- | --- | --- |
| Test score, 0 (no loss of pelvis frontal plane) | • Participant smoothly and easily performs the movement.  • Lower extremities, pelvis, trunk and shoulders remain aligned in the frontal plane. |
| Test score, 1 (minimal loss of pelvis frontal plane) | • Participant may demonstrate a slight wobble at initiation of the movement, but quickly regains control.  • Movement may be performed with noticeable effort or with a slight ratcheting of the moving limb. |
| \|  \| \| --- \| \| Test score, 2 (moderate loss of pelvis frontal plane) \| | • Participant has a noticeable wobble, tipping of the pelvis, rotation of the shoulders or trunk, hip flexion, and/or internal rotation of the abducting limb.  • Movement may be performed too rapidly, and participant may or may not be able to regain control of the movement once it has been lost. |
| Test score, 3 (severe loss of pelvis frontal plane) | • Participant demonstrates the same patterns as in a test score of 2, with greater severity.  • Participant is unable to regain control of the movement and may have to use a hand or arm on the table to maintain balance. |

**Table1**. Scoring criteria of Active Hip Abduction Test (AHAbd)
