# Supplementary material for: The impact of different standing positions on gluteus medius activation and lumbar lordosis in LBP-developers during prolonged standing
Source: PLoS One. 2025 Feb 11;20(2):e0317291. doi: 10.1371/journal.pone.0317291 (PMC11813126; doi:10.1371/journal.pone.0317291)
Supplement: Attachment — Submitted filename: Response to reviewers.docx [file pone.0317291.s005.docx]

**Dear editor and reviewers;**

*We thank you for your insightful and constructive suggestions. Based on your helpful suggestions, we were able to further improve our manuscript. We carefully considered and addressed all your specific comments and revised the text if necessary.*

*Please find your comments in* **bold** *and our responses in italic font. In the manuscript, the highlighted parts in green are the revisions made based on the comments of reviewer1 and the highlighted parts in yellow are the revisions made based on the comments of reviewer2.*

***Thank you for taking the time to provide detailed feedback on our manuscript. We appreciate your insights and constructive criticism. Your comments will be invaluable in refining our study. We carefully address each of the points you have raised to enhance the clarity of our rationale, align the benefits with the actual outcomes, improve the accuracy of our results, and enhance the structure of our discussion. Your input will undoubtedly contribute to the overall quality of our work.***

**Reviewer #1**

**Introduction**

**1* The current introduction section conflates multiple factors, resulting in a slightly weaker logical context for the study. The description of non-critical factors (e.g., excessive VAS discussion) should be simplified to focus more on the characteristics of the PD population versus the core issue of standing posture interventions.**

*Thank you for your comment. We added dedicated paragraphs discussing the critical role of Gmed muscle co-activation, lumbar lordosis, footrest, and arm position during standing, highlighting its relevance to PDs and its implications for LBP development.*

**2*Lack of background on some of the core literature, especially the key studies on the relationship between lumbar lordosis angle and LBP, did not provide enough detailed discussion.**

*Thank you for your comment. We expanded the discussion on the relationship between lumbar lordosis and LBP, referencing specific studies and linking these findings to PD characteristics and interventions:*

*Lumbar lordosis is another significant factor in LBP development during prolonged standing. Research indicates that PDs exhibit significantly greater lumbar lordosis than non-PDs, with a strong correlation between lordosis severity and LBP prevalence [31,32]. During prolonged standing, spinal posture may interact with the stresses placed on the spine, increasing the risk of developing LBP symptoms. These findings are further supported by the significant positive relationship between symptom severity and the degree of lordosis observed, where greater lordosis was associated with greater severity of LBP symptoms, even with acute and transient symptoms during the standing test [31].*

**3*The current introduction is not specific enough in describing the research gaps and fails to clearly state the specific areas where the current literature is lacking. It is recommended that the innovations of the study be more clearly articulated.**

*Thank you for your comment. We Clearly articulated the gaps in the literature, emphasizing the lack of studies on combined interventions (e.g., footrest height and arm positions) for alleviating pain in PDs:*

*Since lumbar lordosis plays a pivotal role in the development of LBP, and prior research highlights the significant impact of arm positioning on lordosis, the lack of studies addressing this relationship in PDs constitutes a notable research gap. Moreover, footrest interventions have been shown to effectively reduce the activity of the Gmed and lumbar muscles in PDs. This study investigates the combined effects of arm positioning and footrest usage during one hour of standing to improve lumbar-pelvic stability, alleviate pain intensity, and offer practical, generalizable solutions for reducing LBP risks in occupations requiring prolonged standing.*

**4* Lines 57-59 refer to “task type” affecting Gmed co-activation, but do not account for its direct association with PD or LBP.**

*Thank you for your notice. We expanded the discussion to explicitly link task type to Gmed muscle co-activation and its potential role in LBP and PDs, as highlighted in relevant studies:*

*Notably, task type during standing directly influences Gmed co-activation and may affect PDs' susceptibility to LBP. For example, tasks involving upper limb activity, such as assembly or sorting, tend to increase Gmed co-activation, while tasks without upper limb movement reduce it [30]. This association highlights the importance of investigating how task-specific factors influence muscle activation patterns in PDs. This association is important because increased Gmed co-activation helps maintain lumbar stability and prevent LBP. Inadequate or inconsistent Gmed activation, especially during dynamic tasks, may increase vulnerability to LBP in PDs.*

**5* The objectives of the study in lines 91-94 should be more specific and should make a natural transition with the logic of the preceding text to avoid abruptness.**

*Thank you for your notice. We refined the study objectives for specificity and ensured they follow logically from the preceding discussion :*

*Since lumbar lordosis plays a pivotal role in the development of LBP, and prior research highlights the significant impact of arm positioning on lordosis, the lack of studies addressing this relationship in PDs constitutes a notable research gap. Moreover, footrest interventions have been shown to effectively reduce the activity of the Gmed and lumbar muscles in PDs. This study investigates the combined effects of arm positioning and footrest usage during one hour of standing to improve lumbar-pelvic stability, alleviate pain intensity, and offer practical, generalizable solutions for reducing LBP risks in occupations requiring prolonged standing.*

**6* The description of “suitable height condition” in line 84 is not supported by clear data and it is recommended that the appropriate experimental background be added.**

*Thank you for your notice. We added supporting experimental evidence from cited studies :*

*So, adjusting footrest height and usage duration is crucial for achieving better outcomes [16,47]. A footrest height equivalent to 10% of body height or adjusted to achieve a 135-degree trunk-to-thigh angle has been shown to reduce lumbar load, fatigue, and pain intensity [44,45]. These devices, combined with optimized upper limb positions, may improve lumbopelvic alignment and alleviate discomfort during prolonged standing.*

**Method**

**7* Some of the sentences are lengthy and contain grammatical errors (e.g., line 125, “participants ‘informed consent’ is not clear), and the descriptions are repetitive.**

*Thank you for your notice. I refined sentences and clarified but the excessive data in this paragraph refer to the editor’s comments that were added, so I cannot remove some of this information:*

*Informed consent was obtained from participants for their involvement in the study and for the publication of identifiable information or images in an open-access online journal. During data collection, access to personally identifiable information was restricted based on this consent, and all data were anonymized for analysis.*

**8* No mention was made of adjusting for confounding variables (e.g., height, weight, etc.) or stratifying the analysis. In addition, the ANOVA model lacked explicit assumptions and explanations, although it mentioned time and condition interactions.**

*Thank you for your notice. Participants with a body mass index (BMI) greater than 30 were excluded. The BMI range of the included participants was between 18 and 22, and their heights were also within a similar range. Therefore, due to the homogeneity of these variables within the sample, their potential influence as confounding variables was negligible. This matter refined in manuscript as bellow:*

*Also ,participants were excluded if they had employment involving standing in one place for more than 1 hour per day in the past 12 months, were unable to stand for more than 4 hours, had a body mass index (BMI) greater than 30, or reported any symptoms of LBP at the start of the standing task.*

**Data Analysis**

**9* Lines 182: There is a lack of detailed description of MVC and the details of solving the entire muscle activation calculation process. Please add the relevant content, the authors may consider referring to: Accurately and effectively predict the ACL force: Utilizing biomechanical landing pattern before and after-fatigue (https://doi.org/10.1016/j.cmpb.2023.107761); New insights optimize landing strategies to reduce lower limb injury risk (**<https://doi.org/10.34133/cbsystems.0126>**)**

*Thank you for your valuable comment. We have revised the manuscript to include a detailed description of the MVC normalization process and the muscle activation calculation. Specifically, we have added the following details:*

- *The procedure for obtaining MVC values through maximum isometric contractions, including the duration and method of calculation.*
- *The normalization of EMG signals to %MVC for consistency and comparability across participants and conditions.*
- *A clear explanation of the calculation pipeline, including the use of RMS for filtered EMG data and further analysis conducted in MATLAB.*

*For normalization, the electromyographic signals were processed by subtracting resting activation levels and then normalizing to maximum voluntary contraction (MVC). MVC values were obtained by asking participants to perform maximum isometric contractions against resistance for each targeted muscle, following standard testing procedures as described in [reference specific source]. These contractions were held for 5 seconds, with the middle 3 seconds of the contraction used to compute the MVC[24]. The root mean square (RMS) of the filtered raw data was then calculated for subsequent analysis. This normalization ensured that the muscle activation data were expressed as a percentage of MVC (%MVC), allowing for comparisons across participants and conditions. Raw EMG data were imported directly into a custom MATLAB (V.4) script for further processing, which included computing the time-domain and frequency-domain characteristics of the muscle activation.*

**10* The article has a relatively small set of indicators, and more data indicators could be added to better explore the research questions.**

*We appreciate your insightful comment. While our study aimed to prioritize key indicators that are most directly relevant to the research questions, we recognize that adding more indicators could provide deeper insights. We will take this into account in designing future studies and expanding the scope of our analysis*

**Reviewer #2**

**Title**

**1* You have just investigated the activation of the gluteus medius! Therefore, gluteus medius activation should be used instead of pelvic muscle activation.**

*Thank you for your notice. The title has been updated accordingly, as shown below:*

**The Impact of different Standing positions on Gluteus Medius Activation and Lumbar Lordosis in LBP-developers during prolonged standing**

**Abstract**

**2* L26: Use gluteus “medius muscle activity” instead of “muscle activity” in the all manuscript.**

*Thank you for your notice. It has been corrected throughout the entire manuscript to "gluteus medius muscle activity."*

**3* P2: Report exact p-value separately for significant parameters in results section.**

*Thank you for your comment. Because the dataset includes numerous parameters for time, condition, and their interaction (time * condition) across three variables and two groups, only the most important results are reported and we added as bellow:*

*No significant interaction was observed between the groups in right and left Gmed co-contraction index (CCI) (p = 0.14). However, both groups exhibited a significant decrease in CCI during prolonged standing (time * condition: p = 0.003). Additionally, Group B consistently demonstrated lower overall levels of co-contraction across time (p = 0.01). An approximate 6-degree reduction in lumbar lordosis was observed after prolonged standing with both interventions (group A and group B; p = 0.008 and p = 0.01, respectively), although no significant differences in lumbar lordosis were detected between the groups. Lumbar discomfort increased over time; however, the interventions significantly alleviated this discomfort after the intervention time point. Notably, group B reported lower pain intensity compared to group A (p = 0.007).*

**4* P2: You must also specify the type of study as well as the statistical test used.**

*Thank you for your notice. We added this sentences as below:*

*These variables were analyzed using repeated measures (between/within) ANOVA.*

**Introduction**

**5* P3: The bilateral co-activation of Gmed was one of the dependent variables in your study, but you did not mention any related articles to indicate its importance in PD or LBP.**

*Thank you for your notice. We* *added a dedicated paragraph discussing the critical role of Gmed muscle co-activation in lumbar and pelvic stability during standing, highlighting its relevance to PDs and its implications for LBP development*:

*The bilateral co-activation of the gluteus medius (Gmed) muscle plays a critical role in maintaining pelvic and lumbar stability during standing. Impaired or altered Gmed activation has been linked to LBP in PDs, contributing to changes in motor control and muscle co-activation [8,12]. It is believed that this co-activation compensates for the inability to properly control the trunk by the core muscles for postural stability [9,26,27]*. *Additionally, the low strength and endurance of this muscle are significantly associated with increased co-activation during prolonged standing [28].*

**6* L 87: What did you mean by “smaller anterior-posterior height ratio”? It is unclear!**

*Thank you for your comment. We rephrased this to clearly describe:*

*Women generally exhibit greater lumbar flexion (a smaller anterior-posterior height ratio of intervertebral discs) compared to men, who demonstrate greater lumbar extension (a larger ratio).*

**Methods**

**7* P6: Please explain how did you estimate the sample size?**

*Thank you for your comment. We clarified as bellow:*

*The sample size was determined using G*Power software version 3.1. With a confidence level of 0.95, an effect size of 0.83[24], and a test power of 0.85, the minimum required sample size was calculated to be 8 participants. However, considering that previous studies included at least 12 participants, the test groups were set to 12 participants each (a total of 24 participants).*

**8* P6: You mentioned that there were two groups; however, there is no information provided regarding the demographics of each group separately or a comparison between them. Additionally, please clarify the randomization methods used.**

*Thank you for your valuable feedback. Both groups were entirely similar in terms of demographics, including age, gender, height, and weight, as all participants were PD.*

*Participants were randomly assigned to each group using "a computer-generated randomization list”.*

**9* L108: Please clarify the time of the “prolonged standing”!**

*Thank you for your comment. We clarified as bellow:*

*In the initial step, all participants underwent a 2-hours prolonged standing test to categorize them as either PD or NPD.*

*On a separate day, participants stood for one hour in a confined workspace.*

**10* P 9: Why didn’t you consider the subject's lower extremity length or total height to determine the box height? This difference in subjects' anthropometric properties obviously affects lower extremity angles and muscle activity!**

*Thank you for your comment. The mean height is : 164.35 (SD: 4.76) cm . We clarified as bellow:*

*A 20-cm footrest was used during the standing period. This height was selected to represent 10% of the participants' average body height and to ensure a 135° trunk-to-thigh angle [44,45] for the majority of participants, which ranged between 17 and 20 cm.*

**11* P 9: It is unclear what the duration of the Gmed muscle activity record is. Have you recorded the muscle activation in which position, and for how many seconds?**

*Thank you for your comment. We clarified as bellow:*

*For each five-minute trial, data were segmented into three one-minute windows representing the following conditions: “Condition 1: before stepping,” “Condition 2: right foot on the footrest with/without arm position change,” and “Condition 3: left foot on the footrest with/without arm position change.” These conditions were repeated four times after every 10 minutes of usual standing, over the course of one hour. Muscle activity was recorded during these three one-minute conditions.*

**12* L190: Determine the muscles! Also, why didn’t you consider the quadratus lumborum muscle and its co-activation with the gluteus medius?**

*Thank you for your comment. The quadratus lumborum was not considered in this study as there is limited evidence in the literature regarding its role in the specific context of PDs. Most studies highlight the gluteus medius, especially its bilateral activation, as crucial in these individuals, which is why we focused on this muscle. Also, we clarified as below:*

*The co-contraction index (CCI) was calculated to quantify the level of co-activation between all possible muscle pairs (left and right gluteus medius) using the following equation [57]:*

**13* L 196: What did you mean by joint activity? It is unclear!**

*Thank you for your comment. We clarified as bellow:*

*This technique is commonly employed to evaluate the coordination between two muscles.*

**Results**

**14* Present the difference and confidence intervals**

*Thank you for your comment. We added CI in tables.*

**15* The figures are unclear. You need to specify the unit measurements for each parameter and indicate significant differences (within and between groups difference) in the figures using an asterisk (*).**

*Thank you for your comment. Since the differences between the two groups were not statistically significant, no asterisks were used in the figures to denote between-group comparisons. However, significant within-group differences were marked with asterisks to highlight those findings in tables.*

**16* There is no figure or table regarding the lumbar lordosis angle.**

*Thank you for your comment. We added a table (* **Table 2.***)*

**Discussion**

**17* Better justification of the study research and inferences you draw for recommending these results.**

*Thank you for your comment. We have revised the discussion to better justify the research and its implications based on your feedback.*
