# Supplementary material for: The impact of different standing positions on gluteus medius activation and lumbar lordosis in LBP-developers during prolonged standing
Source: PLoS One. 2025 Feb 11;20(2):e0317291. doi: 10.1371/journal.pone.0317291 (PMC11813126; doi:10.1371/journal.pone.0317291)
Supplement: Attachment — Submitted filename: Response_to_reviewers_auresp_2.docx [file pone.0317291.s006.docx]

**Dear editor and reviewers;**

***We thank you for your insightful and constructive suggestions. Based on your helpful suggestions, we were able to further improve our manuscript. We carefully considered and addressed all your specific comments and revised the text if necessary.***

*Please find your comments in* **bold** *and our responses in italic font. In the manuscript, the highlighted parts in yellow are the revisions made based on the comments of reviewer1 .*

**Reviewer #1**

1. **The authors mentioned the relationship between low back pain (LBP) and prolonged standing and specific job tasks in the context of the study, but the current literature review seems to be insufficient. It is suggested that the author further explore the latest research progress on the pathogenesis of LBP in recent years, especially the cutting-edge theories related to muscle activation pattern, lumbar stability and task specificity. This helps to provide the reader with richer background information and strengthens the theoretical support of research.**

*Thank you for your comment. We revised the introduction paragraphs as below:*

*The etiology of LBP involves complex interactions between various factors, including anthropometric, structural, psychological, postural, and muscular aspects [6,10–17]. Reports suggest that individuals prone to standing-induced LBP, or Pain Developers (PDs), frequently exhibit biomechanical and motor control deficits. For instance, PDs demonstrate lower Gluteus Medius (GMed) endurance during side-bridge tests, often accompanied by reduced strength [5,18,19].* *The bilateral co-contraction of Gmed muscle plays a critical role in maintaining pelvic and lumbar stability during standing. Impaired or altered Gmed activation has been linked to LBP in PDs, contributing to changes in motor control and muscle co-contraction [20,21]. It is believed that this co-contraction compensates for the inability to properly control the trunk by the core muscles for postural stability [12,22,23]. The active hip abduction (AHAbd) test is often used to identify these deficits, as it predicts standing-induced LBP with high accuracy (0.83) [21,24,25].* *Furthermore, task-specific factors, such as upper limb activity during standing, significantly influence Gmed co-contraction, with implications for LBP development. Tasks requiring upper limb movement, like sorting or assembly, change Gmed co-contraction, potentially reducing trunk instability and LBP risks [26]. Inadequate or inconsistent GMed activation, especially during dynamic tasks, may increase vulnerability to LBP in PDs. Lumbar lordosis is another pivotal factor in LBP development. PDs generally exhibit greater lumbar lordosis compared to non-PDs, with higher lordosis severity correlating with increased LBP symptoms [27,28]. One study noted a significant correlation with maximal visual analog scale (VAS) scores [28]. This association underscores the interplay between spinal posture and mechanical stress during prolonged standing. Research further links lumbar lordosis severity to negative sagittal vertical axis (SVA) displacement, indicating that postural deviations may exacerbate LBP symptoms [28–30]. Gender differences further complicate the understanding of LBP in PDs.* *Women generally exhibit greater lumbar flexion (a smaller anterior-posterior height ratio of intervertebral discs) compared to men. Women also tend to have higher endurance but reduced force capacity, suggesting gender-specific contributions to LBP susceptibility and motor control adaptations [31,32].*

2. **In describing the research method, although the author mentioned the calculation of the MVC and the normalization processing of muscle activation data, the detailed implementation details of these steps and the verification process were not detailed enough. It is recommended that the author add detailed steps about the MVC measurement method, including the equipment used, the measurement position, the posture requirements when measuring, etc., and explain why this method was chosen for MVC measurement. At the same time, for the normalization of muscle activation data, the author should provide detailed instructions on how to screen and filter the original data, how to calculate the root mean square value (RMS) and how to perform the normalization processing and attach the relevant formulas to increase the transparency and repeatability of the study.**

*We appreciate your suggestion. We expanded the method as below:*

*Maximum voluntary contraction (MVC) values were measured by instructing participants to perform maximal isometric contractions against resistance. To normalize and compare muscle activity relative to each muscle's maximal strength, MVCs were recorded for the left and right gluteus medius (Gmed). Participants performed hip abduction in a side-lying position, with resistance applied at the ankle by the experimenter, to obtain MVCs [55,56]. The experimenter can actively monitor and adjust the resistance applied, ensuring participants are performing maximal efforts without exceeding their limits, which could lead to injury. Each leg underwent two sets of MVCs, with a minimum of 30 seconds of rest between efforts. Additionally, 10-second resting trials were conducted with participants lying in both the supine and prone positions [3].*

*Each EMG linear envelope was normalized as a percentage of the maximum voluntary contraction (MVC). Subsequently, the root mean square (RMS) of the filtered raw data was calculated to determine the average intensity of the signal. This was achieved by averaging the squared values using the formula 1/N∑ xi^2^. The normalized muscle activation data were expressed as a percentage of MVC using the formula (RMS EMG/ MVC RMS)*100. This normalization facilitated comparisons across participants and conditions [31].*

3. **When presenting the results of the study, the author mainly described the changes in muscle activation patterns under different conditions. However, the authors' analysis of the mechanisms behind these changes does not seem to go far enough. It is suggested that the authors combine the biomechanical and physiological principles to further explore how these changes affect the stability of lumbar spine and the mechanism of LBP. For example, it is possible to analyze the relationship between changes in muscle activation patterns in different work tasks and lumbar load, and how these changes affect the stress distribution and stability of the lumbar spine.**

*Thank you for pointing this out. We revised the discussion according to your comment as shown in the highlighted parts:*

*The arm position alteration, involving shoulder flexion with hands crossed on the clavicles may influence upper body muscle activation, indirectly affecting lumbar alignment through kinetic chain interactions. Given that the weight of the arms increases the torque on the spine when moving forward and requires increased back muscle strength to maintain balance, this increase in muscle strength affects the fluctuation of body posture and consequently changes lumbo-pelvic parameters, including the amount of activity of the trunk muscles in stabilizing the back during standing [33]. Also, using footrest can cause slight lumbar flexion, reduced lumbar torque, and decreased lumbar erector spinae muscle activity [42].*

*These findings suggest that footrest use and arm position adjustments optimize biomechanical alignment, alleviate lumbar strain, and enhance postural stability during prolonged standing. Moreover, engaging the upper extremities through core muscle activation can reduce Gmed's compensatory role in trunk control and postural stability [12,22,23]. This reduction in Gmed muscle strain during intermittent foot elevation and changing arm position may serve to alleviate some of the discomfort LBP.*

*It is conceivable that altering arm positioning helps decrease lumbar extension demands, allowing for reduced lumbar muscle activation and alleviating muscle fatigue [38,59]. This adjustment not only contributes to pain relief but also underscores the role of strategic muscle activation as a stabilizing mechanism for prolonged standing and reducing the risk of lumbar instability and LBP.*

4. **The author summarizes the research findings in conclusion but mentions the future research direction in a relatively general way. It is suggested that the authors propose more specific and in-depth future research topics based on the research results. For example, the risk of LBP could be further reduced by optimizing job task design, improving the work environment, or introducing specific rehabilitation training methods. At the same time, the authors may also consider combining the results of this study with research in other related fields to form a more comprehensive research perspective.**

*Thank you for your notice. We expanded the conclusion as below:*

*These findings have practical implications for designing workplace interventions to reduce musculoskeletal strain and improve comfort for individuals required to stand for extended periods in various occupational settings. One potential avenue is improving the work environment—such as adjusting workstation height, providing adjustable footrests, or ensuring proper ergonomic setups—could offer further relief by promoting better spinal alignment and reducing muscle fatigue. Future research should explore the long-term effects of these interventions and investigate other potential combinations of more movements and positions to further enhance their effectiveness.*

5. **Although the author has already mentioned some limitations in the study, I think the discussion on methodological limitations could go further. For example, authors may explore the impact of sample size, study design (such as the lack of randomized controlled trials), measurement errors, and other factors on study results, and suggest possible solutions or improvements. This helps readers gain a more complete understanding of the limitations of the study and stimulates more thinking about how to improve the research methodology.**

*Thank you for pointing this out. We refined as below:*

*While the study provides compelling preliminary evidence, several methodological limitations need to be considered. One of the main limitations is the small sample size, which limits the generalizability of the findings. A larger, more diverse sample would allow for better representation of various populations, particularly those with occupational standing requirements. Moreover, the lack of long-term follow-up limits the ability to assess the sustained effects of the interventions. Future studies should include longitudinal designs to examine whether the benefits observed in this study can be maintained over time and how they influence long-term musculoskeletal health.*
