# Supplementary material for: The impact of different standing positions on gluteus medius activation and lumbar lordosis in LBP-developers during prolonged standing
Source: PLoS One. 2025 Feb 11;20(2):e0317291. doi: 10.1371/journal.pone.0317291 (PMC11813126; doi:10.1371/journal.pone.0317291)
Supplement: Attachment — Submitted filename: Reviewer Comment.docx [file pone.0317291.s004.docx]

**Manuscript number** …

**Title** The Impact of different Standing positions on Pelvic Muscle Activation and Lumbar

Lordosis in LBP-developers during prolonged standing

Thank you for choosing me as a reviewer.

**General comment:**

Congratulations it was a good manuscript. But there are several critical points that need to be made clear.

**Title**
- You have just investigated the activation of the gluteus medius! Therefore, gluteus medius activation should be used instead of pelvic muscle activation.

**Abstract**

L26: Use gluteus “medius muscle activity” instead of “muscle activity” in the all manuscript.

P2: Report exact p-value separately for significant parameters in results section.

P2: You must also specify the type of study as well as the statistical test used.

**Introduction**

P3: The bilateral co-activation of Gmed was one of the dependent variables in your study, but you did not mention any related articles to indicate its importance in PD or LBP.

L 87: What did you mean by “smaller anterior-posterior height ratio”? It is unclear!

**Methods**

P6: Please explain how did you estimate the sample size?

P6: You mentioned that there were two groups; however, there is no information provided regarding the demographics of each group separately or a comparison between them. Additionally, please clarify the randomization methods used.

L108: Please clarify the time of the “prolonged standing”!

P 9: Why didn’t you consider the subject's lower extremity length or total height to determine the box height? This difference in subjects' anthropometric properties obviously affects lower extremity angles and muscle activity!

P 9: It is unclear what the duration of the Gmed muscle activity record is. Have you recorded the muscle activation in which position, and for how many seconds?

L190: Determine the muscles! Also, why didn’t you consider the quadratus lumborum muscle and its co-activation with the gluteus medius?

L 196: What did you mean by joint activity? It is unclear!

**Results**:

- Results: Present the difference and confidence intervals

- The figures are unclear. You need to specify the unit measurements for each parameter and indicate significant differences (within and between groups difference) in the figures using an asterisk (*).

- There is no figure or table regarding the lumbar lordosis angle.

**Discussion**

- Better justification of the study research and inferences you draw for recommending these results.
